# Treatment of COVID-19 in Hemodialysis Patients Using Traditional Chinese Medicine: A Single-Center, Retrospective Study

**DOI:** 10.3389/fphar.2022.764305

**Published:** 2022-03-24

**Authors:** Wei Huang, Bo Jiang, Jinli Luo, Meng Luo, Xiaoming Ding, Qian Yang, Lin-Hua Zhao, Qin-Guo Sun, Xiao-Lin Tong

**Affiliations:** ^1^ Wuhan Third Hospital, Tongren Hospital of Wuhan University, Wuhan, China; ^2^ Guangzhou University of Chinese Medicine, Guangzhou, China; ^3^ Guang’anmen Hospital, China Academy of Chinese Medical Science, Beijing, China

**Keywords:** hemodialysis, SARS-CoV-2, COVID-19, Chinese medicine, Western medicine

## Abstract

**Background:** To explore the effect of combining traditional Chinese medicine (TCM) and Western medicine in hemodialysis patients with coronavirus disease 2019 (COVID-19).

**Methods:** This study was conducted from 27 January 2020 to 17 March 2020 in Wuhan Third Hospital Guanggu Branch, Wuhan, China. Fifty-three patients were included and divided into a control group (CG), which received Western medicine and a combined treatment group, which received TCM and Western medicine (TG). Clinical and laboratory data, TCM symptom scores, and chest computed tomography results were extracted and compared between the two groups.

**Results:** The TG included 21 (67.7%) men and 10 (32.3%) women with a mean age of 61.02 (standard deviation [SD] 15.07, range 26–89) years. The mean dialysis duration in the TG was 49 (SD 31) months. Of all patients in the TG, 27 (87.1%) had fatigue, 18 (58.1%) had dry cough, 16 (51.6%) had anorexia, 11 (35.5%) had dyspnea, and 11 (35.5%) had fever. The CG included 14 (63.6%) men and 8 (36.4%) women with a mean age of 61.45 (SD 13.78, range 36–84) years. The mean dialysis duration in the CG was 63 (SD 46) months. Of all patients in the CG, 21 (95.5%) had fatigue, 12 (54.5%) had dry cough, 17 (77.3%) had anorexia, 12 (54.5%) had dyspnea, and 7 (31.8%) had fever. After treatment, the TCM symptom scores of the two groups decreased; the anorexia scores were lower in the TG than in the CG (*p* < 0.05). After treatment, albumin increased and D-dimer, C-reactive protein, and lactate dehydrogenase levels decreased in the TG. The d-dimer levels were lower and the albumin level was higher in the TG than in the CG after treatment (*p* < 0.05). The cure rate was higher, and the mortality rate was lower in the TG than in the CG (*p* < 0.05).

**Conclusion:** A combination of TCM and Western medicine in hemodialysis patients with COVID-19 could relieve symptoms and help recovery. Further evidence from larger randomized controlled trials is needed to confirm our results.

## Introduction

Coronavirus disease 2019 (COVID-19), which was first reported in December 2019, has become a global pandemic, resulting in considerable morbidity and mortality in 187 countries and regions worldwide, especially in patients with underlying diseases, such as kidney diseases (Naicker et al., 2020). Previous studies reported that 2.15–11% patients receiving maintenance hemodialysis (HD) were susceptible to COVID-19, and HD centers have been high-risk settings during the epidemic in China (Xiong et al., 2020; Zou et al., 2020). Because of the large number of patients visiting the HD centers, there is an increased risk of severe acute respiratory syndrome coronavirus 2 (SARS-CoV-2) infection.

Traditional Chinese medicine (TCM) has accumulated experience of several decades in the treatment of pandemic and endemic diseases with multi-component, multi-target, and multi-effect modes of action. Currently, TCM is frequently used in combination with Western medicine for the treatment of kidney disease and has shown multiple treatment effects with low toxicity and few adverse effects. An open-label trial reported that Ren Shen Yang Rong Tang could decrease chronic inflammation and increase the quality of life in HD patients (Hsiao et al., 2015). In addition, TCM uremic clearance granule could significantly relieve clinical symptoms, reduce the level of inflammatory factors and antioxidative stress, and have a certain effect on renal anemia, calcium and phosphorus metabolism disorders, and other complications (Bai et al., 2021).

The association between HD-associated COVID-19 and the risk of mortality is unclear. Our study retrospectively analyzed data from Wuhan Third Hospital Guanggu Branch, Wuhan, China. We aimed to describe the clinical, laboratory, and radiological characteristics; TCM symptom scores; treatment; and outcomes in HD patients confirmed to have SARS-CoV-2 infection, and to compare these factors in patients who received Western medicine and those who received a combination of Western medicine and TCM. We hope our study will provide the global community with information about clinical features and treatment of hemodialysis-associated COVID-19.

## Methods

### Study Design and Participants

This non-randomized, single-center, retrospective study was conducted at Wuhan Third Hospital Guanggu Branch (Wuhan, China), which is a hospital designated to treat patients with SARS-CoV-2 pneumonia. We retrospectively analyzed 53 patients who had been diagnosed with SARS-CoV-2 pneumonia from 27 Jan 2020 to 17 Mar 2020. Based on the intervention method, 22 patients who received Western medicine were enrolled in the control group (CG) and 31 patients who received a combination of Western medicine and TCM were enrolled in the treatment group (TG).

This study was approved by the ethics commission of Wuhan Third Hospital (KY-2020-016). Written informed consent was waived by the ethics commission of the designated hospital.

### Data Collection

The demographic characteristics (age and sex), clinical data (symptoms, comorbidities, laboratory findings, treatments, complications, and outcomes), and TCM symptom scores were collected. Radiological assessments included chest computed tomography (CT). The clinical outcomes (i.e., discharges, mortality, and length of stay) were monitored up to 17 March 2020, which was the final date of follow-up. All data were evaluated by two physicians (WH and JL) and a third researcher (BJ) adjudicated any differences in interpretation between the two primary reviewers.

### Eligibility Criteria and Treatment

Broad eligibility criteria were used to increase the generalizability of the study. The inclusion criteria were as follows:

1. A diagnosis of COVID-19 based on the Western medicine diagnostic criteria according to the Chinese management guidelines for COVID-19 (version 5.0) (National Health Commissio, 2020), which include the following:

a) epidemiological history, clinical manifestations of fever and/or respiratory symptoms, CT scan showing features of pneumonia, and RT-PCR (Real-time polymerase Chain Reaction) positive for SARS-CoV-2 nucleic acid in nasopharyngeal swab specimens. Moderate cases were defined as those with fever, respiratory symptoms, and radiological manifestations of pneumonia. Severe cases were defined when one of the following conditions was met: (a) respiratory distress: respiratory rate ≥30 times/min, (b) hypoxemia: oxygen saturation ≤93%, or (c) arterial blood oxygen partial pressure/oxygen concentration ≤300 mmHg (1 mmHg = 0.133 kPa). Critical cases were defined when one of the following conditions was met: (a) respiratory failure requiring mechanical ventilation, (b) shock, or (c) combination of other organ failures requiring intensive care unit monitoring.

2. A diagnosis of COVID-19 based on the Chinese medicine diagnostic criteria according to the Chinese management guidelines for COVID-19 (version 5.0) (National Health Commissio, 2020), which include the following:

(a) cold-damp obstruction of the lung pattern, characterized by (i) low-grade fever, unsurfaced fever, or no fever; (ii) dry cough with little sputum; (iii) lassitude and fatigue; (iv) chest tightness; (v) stomach discomfort or nausea, and loose stools; (vi) pale or light red tongue with a white or white greasy coating; and (vii) a soggy pulse. The recommended formula for this includes Cang Zhu (Atractylodes macrocephala Koidz.) 15 g, Chen Pi (Citrus × aurantium L.) 10 g, Hou Po (Magnolia officinalis Rehder and E.H.Wilson) 10 g, Huo Xiang (Pogostemon cablin [Blanco] Benth) 10 g, Cao Guo (Lanxangia tsao-ko [Crevost and Lemarié] M.F.Newman and Skornick) 6 g, ShengMa Huang (Ephedra sinica Stapf [EH]) 6 g, Qiang Huo (Hansenia weberbaueriana [Fedde ex H. Wolff] Pimenov and Kljuykov) 10 g, Sheng Jiang (Zingiber officinale Roscoe) 10 g, and Bing Lang (Areca catechu L.) 10 g.
(b) the epidemic toxin-blocking lung pattern, characterized by (i) fever with a red face; (ii) cough with little yellow and sticky sputum, or blood-stained sputum; (iii) chest tightness and shortness of breath; (iv) lassitude; (v) dryness, bitterness, and stickiness in the mouth; (vi) nausea and loss of appetite; (vii) difficulty in defecation; (viii) scanty dark urine; (ix) a red tongue with a yellow greasy coating; and (x) a slippery and rapid pulse. The recommended formula for this includes Sheng Ma Huang (EH) 6 g, Xing Ren (Prunus armeniaca L.) 9 g, Sheng Shi Gao (Gypsum fibrosum) 15 g, Gan Cao (Glycyrrhiza glabra L.) 3 g, Huo Xiang (Pogostemon cablin [Blanco] Benth; added later) 10 g, Hou Po (Magnolia officinalis Rehder and E.H.Wilson) 10 g, Cang Zhu (Atractylodes macrocephala Koidz.) 15 g, Cao Guo (Lanxangia tsao-ko [Crevost and Lemarié] M.F.Newman & Skornick.) 10 g, Fa Ban Xia (Pinellia ternata [Thunb.] Makino) 9 g, Fu Ling (Carapichea ipecacuanha [Brot.] L.Andersson) 15 g, Sheng Da Huang (Rheum palmatum L.; added later) 5 g, Sheng Huang Qi (Astragalus mongholicus Bunge) 10 g, Ting Li Zi (Descurainia sophia [L.] Webb ex Prantl) 10 g, and Chi Shao (Paeonia lactiflora Pall.) 10 g.
(c) the internal blockage and external desertion pattern, characterized by: (i) dyspnea, (ii) panting on exertion or mechanical ventilation required, (iii) unconsciousness and dysphoria, (iv) sweating, (v) cold extremities, (vi) dark and purple tongue with a thick greasy or dry coating, and (vii) a floating large tongue without root. The recommended formula for this includes Take Su He Xiang Wan or Angong Niuhuang Wan with the following decoction composed of Ren Shen (Panax ginseng C.A.Mey.) 15 g, Hei Shun Pian (Aconitum carmichaeli Debeaux) (decocted first) 10 g, and Shan Zhu Yu (Cornus officinalis Siebold and Zucc.) 15 g.

3. A diagnosis of end-stage renal disease (ESRD) defined according to the National Kidney Foundation-Kidney/Dialysis Outcomes Quality Initiative (NKF-K/DOQI) (Ureña, 2008), with glomerular filtration rate <15 ml/min/1.73 m^2^, accompanied by symptoms and signs of uremia, or administration of kidney replacement therapy (dialysis or kidney transplantation).

The exclusion criteria were as follows:

1) Incomplete diagnosis and treatment information,
2) Final outcome data missing.

### Group Stratification and Interventions

Patients were divided into a CG (*n* = 22) and TG (*n* = 31) based on the type of treatment received. The CG received Western medicine treatment only and the TG received Western medicine treatment and TCM, including one 200 ml dose after decoction divided equally into a 100 ml morning and evening dose, respectively, for 5 days. If after 14 days of treatment, the patient’s symptoms were not significantly relieved, we proposed the combination of Chinese and Western medicine. The patients of the two groups were not in the same ward, and the analysis of outcomes was blinded to treatment allocation.

### Assessment of the Primary Outcomes, Treatment Effect, and Safety

Methods for laboratory confirmation of SARS-CoV-2 infection have been described elsewhere (Huang et al., 2020). Throat-swab specimens were obtained for SARS-CoV-2 PCR re-examination every other day after clinical remission of symptoms, including fever, cough, and dyspnea, but only qualitative data were available. The criteria for discharge were absence of fever for at least 3 days, substantial improvement in both lungs on chest CT, clinical remission of respiratory symptoms, and two throat-swab samples negative for SARS-CoV-2 RNA obtained at least 24 h apart. Routine blood examinations included complete blood count, coagulation profile, serum biochemical tests (including renal and liver function, creatine kinase, lactate dehydrogenase (LDH), electrolytes, C-reactive protein (CRP), and procalcitonin. Chest radiographs or CT scan were performed for all patients. The frequency of examinations was determined by the treating physician.

TCM symptom scores were calculated according to the “Guiding principles for clinical research of new Chinese medicine (the 2010 revision)” (Zheng et al., 2002). Patients were scored according to the TCM symptoms, including fever, fatigue, dry cough, anorexia, dyspnea, and other symptoms using the following scale: none (1 point), light (2 points), medium (3 points), and heavy (4 points). Scores were evaluated before and after the treatment. According to the TCM syndrome score, an effect evaluation was performed. The evaluation criteria were as follows: (Naicker et al., 2020): significantly effective: TCM symptoms and signs were significantly relieved, and the total score was reduced by ≥ 70%, (Xiong et al., 2020), effective: TCM symptoms and signs were relieved, and the total score was reduced by ≥ 30%, and (Zou et al., 2020) invalid: TCM symptoms and signs were basically unchanged, and the total score was reduced by <30%. Two professional Chinese medicine practitioners independently performed the measurement and assessment, and we took the average of the two as the patient’s TCM symptom scores.

The standard criteria for discharge were as follows: (Naicker et al., 2020): return of temperature to normal for more than 3 days, (Xiong et al., 2020), significant improvement in the respiratory symptoms and obvious absorption of inflammatory lesions on chest CT, and (Zou et al., 2020) two consecutive negative respiratory tract nucleic acid tests (sampling time interval of at least 1 day), indicating clinical recovery.

### Statistical Analysis

Descriptive data are expressed as mean (± standard deviation [SD]) for continuous variables and number (%) for categorical variables. We assessed the differences between the CG and TG using a two-sample *t*-test or Wilcoxon rank-sum test depending on parametric or nonparametric data for continuous variables and a Fisher’s exact test for categorical variables. The tests were two-sided with significance set at *α* < 0.05. All statistical analyses were performed using the Statistical Product and Service Solutions software (SPSS 23.0, IBM, Chicago, United States).

## Results

The demographic and baseline clinical characteristics of the patients were similar in the two groups (Table 1).

**TABLE 1:** Baseline clinical characteristics of hemodialysis patients affected by SARS-CoV2 infection.

	Classification	TG	CG	p value
Sex	Male	21	14	0.237
	Female	10	8	0.637
Age, years		61.06 (26–89)	61.45 (36–84)	0.924
Typy	Moderate	19	15	
	Severe	6	3	0.56
	Critical	6	4	
Hypertension(%)		27 (87.1)	19 (86.4)	
Diabetes(%)		7 (22.6)	4 (18.2)	
Coronary heart disease(%)		10 (32.2)	5 (22.7)	
Cerebrovascular disease(%)		3(9.7)	0	0.191
Malignancy(%)		3(9.7)	1 (4.5)	
Chronic obstructive pulmonary disease(%)		1 (3.2)	0	
Chronic hepatitis(%)		1 (3.2)	2 (9.1)	

Symptom severity in the patients at baseline was similar in the two groups (Table 2).

**TABLE 2:** Symptom severity of the hemodialysis patients affected by SARS-CoV2 infection.

Symptom Scores	TG	CG
Fever	1.52 ± 0.77	1.45 ± 0.74
Fatigue	2.23 ± 0.67	2.55 ± 0.74
Dry cough	1.77 ± 0.80	1.82 ± 0.85
Anorexia	1.81 ± 0.91	2.14 ± 0.77
Dyspnea	1.81 ± 0.91	1.95 ± 1.09

**FIGURE 1:**
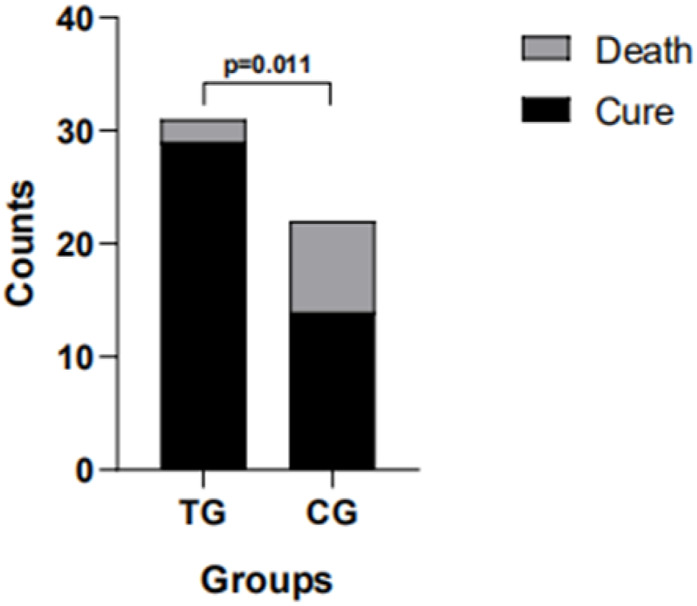
In the treatment group (TG), 29 patients recovered and two died; in the control group (CG), 14 patients recovered and eight died.

Figure 1 shows the outcomes in the two groups. The mortality rate was significantly lower in the TG than in the CG, and the cure rate was significantly higher (p = 0.011). As shown in Table 3, there were 19 moderate cases, six severe cases, and six critical cases in the TG. However, two critical patients died, and patients in all moderate cases and severe cases were cured and discharged, showing that the recovery rate was higher in the moderate and severe groups. Moreover, we found that there were 12 cases of cold-damp obstruction of the lung pattern, 11 cases of epidemic toxin blocking lung pattern, and 7 cases of internal blockage and external desertion pattern in the TG. Two patients with internal blockage and external desertion pattern died, and the other two categories of patients were cured and discharged. This suggests that early Chinese medicine intervention for COVID-19 may be more effective in the treatment and prognosis of patients (Table 4).

**TABLE 3:** Recovery rate of moderate/severe/critical cases in the treatment group.

Typys		Cured	Deaths
Moderate	19	19	0
Severe	6	6	0
Critical	6	4	2

**TABLE 4:** Effect of formula on each diagnostic category in the treatment group.

Typys		Cured	Deaths
Cold-damp obstruction of the lung pattern	12	12	0
Epidemic toxin blocking lung pattern	11	11	0
Internal blockage and external desertion pattern	7	5	2

**FIGURE 2:**
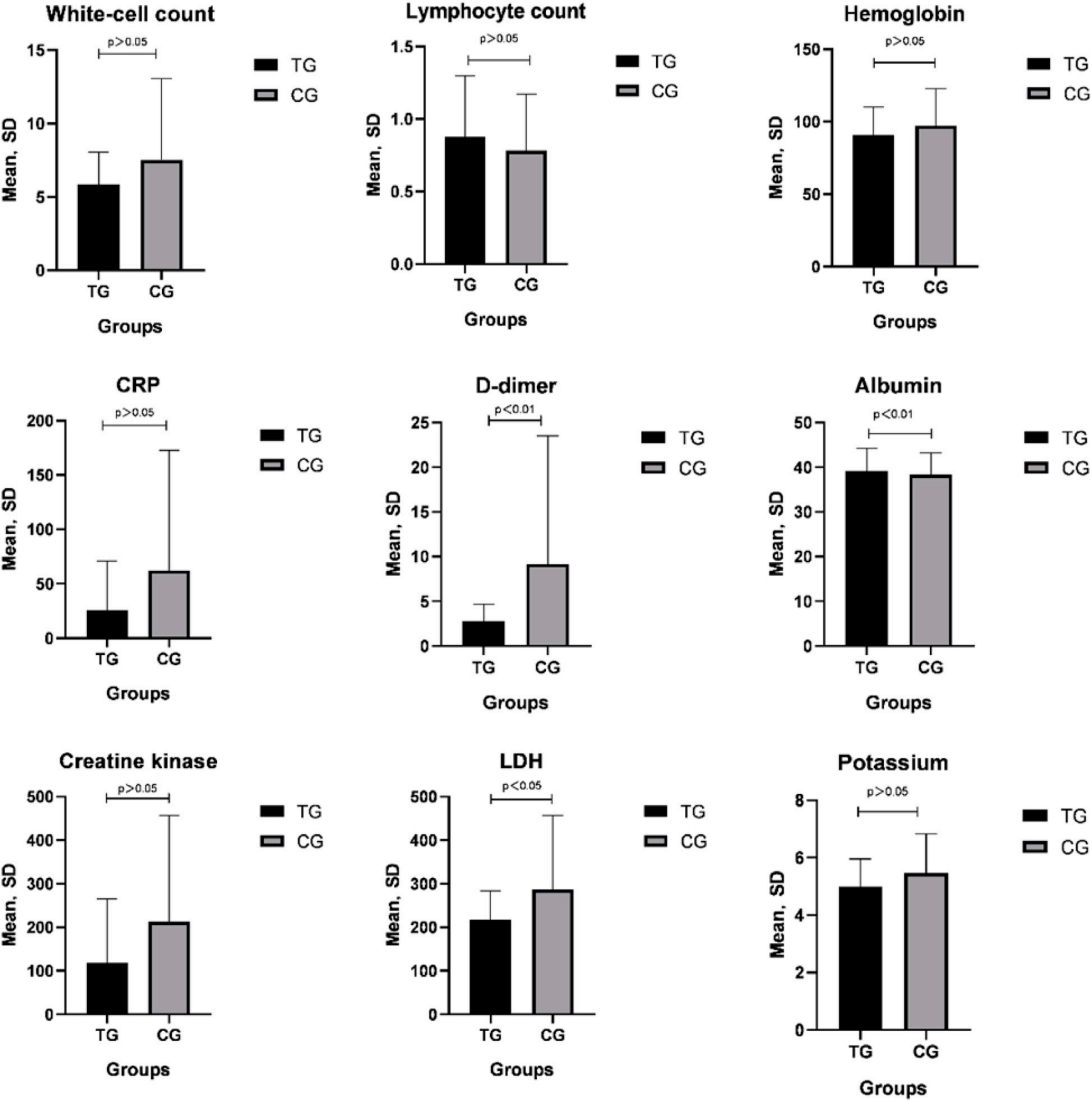
CRP, d-dimer, albumin, and LDH were significantly different compared with pretreatment values in both groups (*p* < 0.05); posttreatment D-dimer values of the TG decreased significantly compared with those in the CG (*p* < 0.05).

`Patients treated with a combination of TCM and Western medicine showed a significantly greater increase in albumin, and a significantly greater reduction in CRP, d-dimer, and LDH than those in the CG. After treatment, the d-dimer levels were lower and albumin was higher in the TG (both p < 0.05) (Figure 2).

**FIGURE 3:**
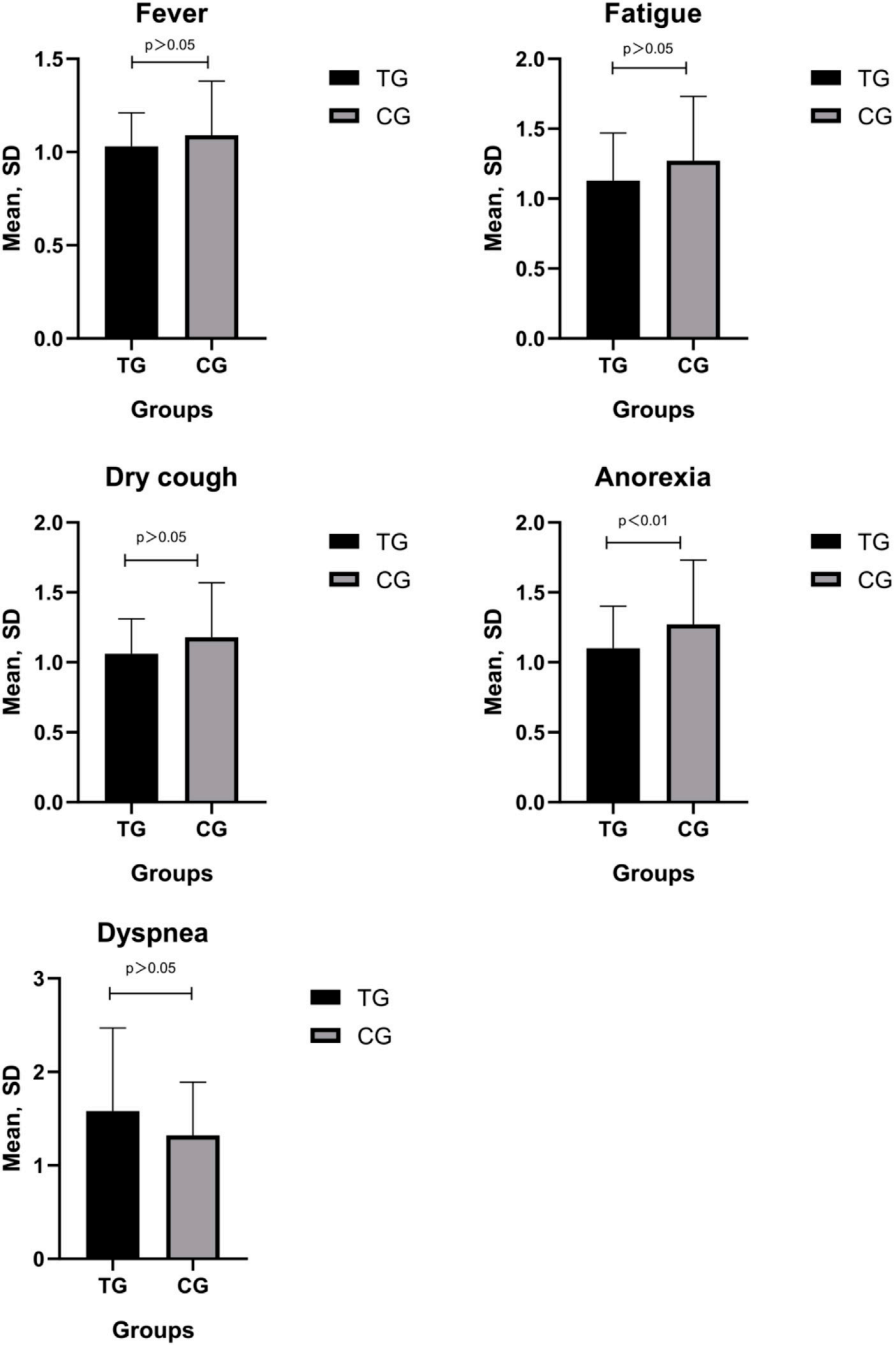
Posttreatment TCM symptom scores were significantly lower compared with the pretreatment scores in both groups (^∇^ *p* < 0.05); The symptom score of anorexia was significantly lower compared with the posttreatment scores in the CG (*p < 0.01).

As shown in Figure 3, TCM symptom scores were significantly reduced after treatment in both groups. The improvement in anorexia was significantly more obvious in the TG than in the CG (p ≤ 0.001).

**FIGURE 4:**
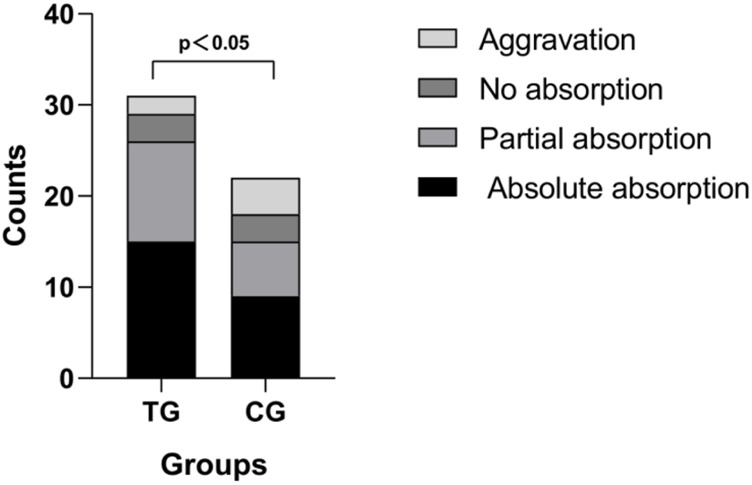
In the treatment group (TG), 15 (48%) cases showed absolute absorption, 11 (36%) showed partial absorption, 3 (10%) showed no absorption, and two showed aggravation (6%); in the control group (CG), 9 (41%) cases showed absolute absorption, 6 (27%) showed partial absorption, 3 (14%) showed no absorption, and 4 (18%) showed aggravation.

Table 5; Figure 4 show the admission chest CT scan findings in the two groups of patients and the CT scan findings on absorption of lung lesions after treatment, respectively.

**TABLE 5:** Admission chest CT scans of the two groups.

	Count	Lesion		Density		Pleural Effusion
		Unilateral	Bilateral	Ground-Glass Opacity	Consolidation	
TG	31	2	29	26	1	15
CG	22	4	18	17	0	3

Table 6 shows clinical adverse events reported in the two groups. Before TCM treatment, four patients had mild liver injury. In terms of liver function (alanine aminotransferase [ALT], aspartate aminotransferase [AST], and direct bilirubin [DBIL]) before and after treatment in the TG, liver function returned to normal after treatment in one patient and remained mild in two patients, and one patient died. The remaining patients were had normal liver function before and after TCM treatment, and their ALT and AST values did not change (p > 0.05). Although there was a significant difference in DBIL before and after treatment in the TG (*p* < 0.05), the indicators were within the normal range, suggesting that there were no serious drug-related adverse reactions during TCM treatment.

**TABLE 6:** Comparison of liver function between the two groups before and after treatment.

	TG ( $\bar{X}$ ± S )		*p* Value	CG ( $\bar{X}$ ± S )		*p* Value
	Pretherapy	Post-treatment		Pretherapy	Post-treatment	
ALT/(U/L)	15.58 ± 7.99	12.84 ± 7.15	0.117	14.56 ± 7.08	14.78 ± 7.61	0.917
AST/(U/L)	18.39 ± 7.91	16.32 ± 6.81	0.144	18.11 ± 7.47	21.28 ± 10.63	0.166
DBIL/(mmol/L)	6.24 ± 3.82	5.36 ± 2.65	0.022	5.28 ± 2.80	5.90 ± 4.48	0.330

## Discussion

SARS-CoV-2 affects the lungs, causing a lingering disease that is difficult to cure (WANG et al., 2020). Some studies (Ji et al., 2020; Shah et al., 2020) have shown that most patients present with a slow onset, long incubation period, and mild symptoms. However, in the later stages of the disease, some patients develop phlegm-stasis, which presents as obstruction of the lung, epidemic toxin-blocking lung, and internal blockage and external desertion patterns on CT. The clinical manifestations are fever, cough, expectoration, fatigue, chest distress, shortness of breath, and other symptoms. Complications include severe pneumonia, acute respiratory failure, and myocardial damage. A previous study (Guan et al., 2020) showed that fever and cough are the main symptoms and vomiting and diarrhea are minor symptoms. In this study, the main clinical manifestations of HD patients with COVID-19 were fatigue, dry cough, anorexia, fever, and dyspnea, which may be difficult to distinguish from other common symptoms of ordinary HD patients.

Albumin, which is synthesized by the liver, is the main serum protein component of the human body, which maintains osmotic pressure and resists infection and oxidation (Caraceni et al., 2016; Zaccherini and Bernardi, 2019). Pretorius et al. (Pretorius et al., 2013) reported that the decrease in serum albumin can increase the risk of infection and thrombosis and cause major cardiovascular adverse events. In our study, the serum albumin level of the patients in the TG was higher than that in the CG after treatment with TCM. The clinical manifestations of HD patients are often fatigue and anorexia, accompanied by anemia. The TCM used in our study improved the clinical symptoms and serum albumin levels; however, further research is needed on the specific mechanisms. In addition, CRP and LDH were lower in the TG after treatment. CRP is the main indicator for evaluating systemic inflammatory response. LDH is an important biochemical indicator of the body’s response to tissue cell hypoxia and blood perfusion, which could be used as a detection index for disease severity and prognosis.

HD patients often show varying degrees of micro-inflammation, which may reduce the patient’s immune function and increase the risk of infection. Xing Ren (Prunus armeniaca L.) and Ma Huang (EH) can promote the synthesis of lung surfactants and have anti-inflammatory, bacteriostatic, and antiviral effects. Wei Xiaolu (Xiaolu, 2013) reported that the main medicinal ingredient of Huo Xiang (Pogostemon cablin [Blanco] Benth.) is patchouli oil, which could act as an anti-adenovirus, probably by destroying viral genes and preventing viral adsorption. It has been confirmed that the immune system dysfunction and chronic infections in ESRD cause multiple complications. Frequent HD causes insufficient blood perfusion in the kidneys, affecting the body’s electrolyte balance, calcium and phosphorus absorption, and toxin clearance (Vanitha et al., 2016). An increase in inflammatory factors in the patient’s serum aggravate the infection, which is obviously related to the severity of the disease. Therefore, it is important to enhance the host’s immunity and suppress the inflammatory response in patients (Kato et al., 2008; Wehmeyer et al., 2013). The Ma Xing Shi Gan Decoction (Hsieh et al., 2012) provides effective anti-inflammatory, anti-influenza, and immune-enhancing effects, and provides oseltamivir-like anti-influenza virus ceramide enzyme activity, and downregulates the secretion and expression of interferon (IFN)-*α/β* in macrophages infected by influenza (Zhang et al., 2019). We found that TCM can effectively reduce CRP and LDH, reduce inflammation, and promote inflammation absorption. The diagnosis and treatment guidelines mention that continuous renal replacement therapy is used to treat critically ill patients to remove cytokines and other inflammatory mediators (Zaccherini and Bernardi, 2019). Hemodialysis can eliminate most inflammatory mediators and cytotoxic substances that cause increased permeability of the blood vessel wall, and this may have affected the improvement in inflammatory indicators in the TG. Uremia is associated with widespread impairment of lymphocyte and granulocyte functions, and an abnormal immune system may change such patients’ response to SARS-CoV infection (Zhou et al., 2017). EH has been used in Asian traditional herbal medicine to cure bronchial asthma, cold, flu, chills, fever, headache, nasal congestion, and cough (Soni et al., 2004). EH contains two main active constituents, ephedrine and pseudoephedrine, which are potent sympathomimetic drugs that stimulate α-, β1-, and β2-adrenoceptors (Ma et al., 2007). However, legal restrictions regarding ephedrine products exist in several Western countries. As of 2004, the US food and drug association received more than 18,000 adverse reports from people using ephedrine to treat asthma, colds, allergies, and various other diseases (Soni et al., 2004). However, EH is one of the herbal medicines from TCM that was successfully used for the treatment of SARS-CoV-infected patients in the 2002/2003 SARS epidemic; thus, the antiviral activity of EH is separable from its adverse effects (Mantani et al., 1999; Liu et al., 2012; Wang et al., 2014; Wei et al., 2019; Luo et al., 2020). Currently, EH extract is ranked third in terms of priority treatment for COVID-19 in China (Oesch et al., 2021).

A study of 1,099 confirmed COVID-19 patients found that among 173 severe patients, 23.7% had hypertension, 16.2% had diabetes, 5.8% had coronary heart disease, and 2.3% had cerebrovascular disease (Guan et al., 2020). We found that hypertension accounts for the highest proportion of chronic underlying diseases, suggesting that patients with hypertension may be more susceptible to SARS-CoV-2. SARS-CoV and SARS-CoV-2 mainly bind to target cells through angiotensin-converting enzyme 2 (ACE2), which can be expressed in the epithelial cells of the lung, intestine, kidney, and blood vessels; promote the expression of ACE2 (Li et al., 2017); and improve the susceptibility of the body to SARS-CoV-2. ACE2 (Wan et al., 2020; Wrapp et al., 2020) seems to be unrelated to infectivity, but it has an impact on disease severity in terms of lung, heart, and kidney function. SARS-CoV-2 infection can affect the lung, myocardium, kidney, liver, digestive tract, pancreas, brain, and other organs, increasing the severity and fatality in COVID-19 patients. However, the above theory is not supported by large-scale clinical data. The latest research shows that the key receptor of the new coronavirus—ACE2—is nearly 100 times higher in the kidneys than in the lungs, especially the proximal tubules (Dai et al., 2020). The kidney may be one of the main targets of the virus, and kidney damage may eventually lead to multiple organ failure and death. However, the characteristics of kidney damage related to the new coronavirus infection need to be investigated further. It has been reported (Yang et al., 2017) that HD can stimulate the ACE/Ang II/AT1R axis (Angiotensin Converting Enzyme/Angiotensin II/Angiotensin Receptor 1) in the circulation of patients with cardiovascular disease, thereby reducing the activity of the ACE2/Ang- (Ureña, 2008; Hsiao et al., 2015; Naicker et al., 2020; National Health Commissio, 2020; Xiong et al., 2020; Zou et al., 2020; Bai et al., 2021)/Mas axis and reducing the concentration of ACE2.

COVID-19 is a major global human threat that has turned into a pandemic. Although specific therapeutic agents or vaccines for COVID-19 are available, this novel coronavirus has specifically high morbidity in elderly and comorbid populations. Therefore, all measures must be taken to decelerate, if not to eradicate the pandemic and to control the unmanageably high incidence rates. According to the clinical manifestations of patients, TCM or integrated Chinese and Western medicine interventions can adjust the body’s immune function and stimulate the body’s ability to resist disease. According to the report of National Administration of Traditional Chinese Medicine, 214 COVID-19 patients in four Chinese provinces were treated with Qing Fei Pai Du Tang with an overall effectivity rate ≥90%. Among them, the symptoms of most patients (≥60%) were markedly improved, while illness of others (30%) was stabilized (Zhong et al., 2020). Thereafter, 701 COVID-19 patients were treated with Qing Fei Pai Du Tang in ten provinces in China. The result showed that 130 patients (18.5%) were completely cured, 268 patients (38.2%) showed improvement in symptoms, and 212 patients stabilized after treatment. In the treatment of confirmed and suspected COVID-19 patients, the Lian Hua Qing Wen Capsule (LHQWC) was shown to markedly relieve major symptoms, such as fever and cough, and promote recovery (Yang et al., 2020). Moreover, LHQWC was shown to significantly inhibit SARS-COV-2 replication, affect virus morphology, and exert anti-inflammatory activity *in vitro* (Runfeng et al., 2020).

SARS-CoV-2 infection presents a special threat to patients on dialysis, and death is deemed to be due to cardiovascular causes and not directly due to the infection. One HD center in Wuhan reported 37 cases of COVID-19 among 230 HD patients (16.1%) and four cases among 33 staff members (12.1%). Dialysis patients with COVID-19 had less lymphopenia, lower serum levels of inflammatory cytokines, and milder clinical disease than other patients affected by SARS-CoV-2 infection (Centers for Disease Contr, 2020). Other studies (Guan et al., 2020; Biarnés-Suñé et al., 2021) reported that elderly patients and patients with comorbidities might be at an increased risk of death if they contract COVID-19. Patients with end-stage renal disease and undergoing maintenance HD are at a greater risk of viral transmission because of impaired immune function and high comorbidity (Dalrymple and Go, 2008; Kato et al., 2008; Butler et al., 2020). Moreover, they are exposed to frequent and repetitive potentially infectious risk factors during regular HD treatment (Sarnak and Jaber, 2000). In this study, each patient was immediately admitted to the isolation ward and received supplemental oxygen through a face mask. The control group was given Arbidol as antiviral therapy, and ceftriaxone sodium/tazobactam sodium injection plus moxifloxacin as antibiotics. Considering renal failure, maintenance HD (two times per week) was administered and restrictions on water intake were enforced to attenuate kidney burden. Albumin and globulin were administered for hypoproteinemia. On the basis of the abovementioned treatment, in addition to the formula recommended in the guide, we also used proprietary Chinese medicines, such as Liuwei Dihuang pills, Huoxiang Zhengqi powder, Jinguishenqi pills, Sijunzi decoction, and according to the patient’s condition in the TG. The mortality rate of the patients in our study was 18.9%. Most patients who died were elderly patients with multiple underlying diseases, such as hypertension, diabetes, coronary heart disease, cerebrovascular disease, and malignancy, and a duration of HD of more than 5 years. Although there was no obvious difference in CT scans between the TG and CG, the cure rate was higher and mortality rate was lower in the TG than in the CG, suggesting that TCM may have a potential role in relieving complications and improving patient survival.

However, the question as to whether patients receiving HD are at a higher risk of mortality from COVID-19 and the mechanisms behind the positive effects of TCM remain to be analyzed in larger clinical studies in future. Further double-blinded, prospective, randomized controlled trials are needed to fully evaluate the effect of TCM in many patients receiving HD.

In summary, TCM and Western medicine both have their own advantages. The integration of TCM and Western medicine might be an effective and feasible solution for curing COVID-19, and more clinical research is needed to confirm this.

## Data Availability

The raw data supporting the conclusion of this article will be made available by the authors, without undue reservation.
